# Comparison of Failure for Thin-Walled Composite Columns

**DOI:** 10.3390/ma15010167

**Published:** 2021-12-27

**Authors:** Patryk Rozylo

**Affiliations:** Department of Machine Design and Mechatronics, Faculty of Mechanical Engineering, Lublin University of Technology, Nadbystrzycka 36, 20-618 Lublin, Poland; p.rozylo@pollub.pl

**Keywords:** failure of composite materials, thin-walled open section structures, progressive failure analysis, cohesive zone model

## Abstract

The novelty of this paper, in relation to other thematically similar research papers, is the comparison of the failure phenomenon on two composite profiles with different cross-sections, using known experimental techniques and advanced numerical models of composite material failure. This paper presents an analysis of the failure of thin-walled structures made of composite materials with top-hat and channel cross-sections. Both experimental investigations and numerical simulations using the finite element method (FEM) are applied in this paper. Tests were conducted on thin-walled short columns manufactured of carbon fiber reinforced polymer (CFRP) material. The experimental specimens were made using the autoclave technique and thus showed very good strength properties, low porosity and high surface smoothness. Tests were carried out in axial compression of composite profiles over the full range of loading—up to total failure. During the experimental study, the post-buckling equilibrium paths were registered, with the simultaneous use of a Zwick Z100 universal testing machine (UTM) and equipment for measuring acoustic emission signals. Numerical simulations used composite material damage models such as progressive failure analysis (PFA) and cohesive zone model (CZM). The analysis of the behavior of thin-walled structures subjected to axial compression allowed the evaluation of stability with an in-depth assessment of the failure of the composite material. A significant effect of the research was, among others, determination of the phenomenon of damage initiation, delamination and loss of load-carrying capacity. The obtained results show the high qualitative and quantitative agreement of the failure phenomenon. The dominant form of failure occurred at the end sections of the composite columns. The delamination phenomenon was observed mainly on the outer flanges of the structure.

## 1. Introduction

Nowadays, there is a high demand for using more modern construction materials. The evolution of the industry determines the use of lightweight and high-strength structures. Composite structures represent an ideal alternative to typical engineering materials. A special group of composite materials is carbon-fiber-reinforced polymer composite materials, used in bridge engineering [1], underground oil fields [2], and civil engineering [3], among others. The phenomenon of failure is of high significance in the analysis of composite materials. The phenomenon of failure, especially in the context of thin-walled composite columns with open sections, constitutes a current engineering issue. The failure process requires the simultaneous use of several independent test methods, allowing for a thorough analysis of the limit states, directly accompanying the failure of the composite material [4,5,6,7,8,9,10]. In order to allow for detailed investigations in the context of the behavior of thin-walled composite columns, it is necessary to observe the behavior of the structure over the full range of compressive loading. During the initial axial loading phase of the structure, there is only compression of the walls of the structure, without the appearance of additional deflections. Further axial compression of the structure leads directly to the phenomenon of loss of stability (buckling) [11,12,13]. Once the buckling phenomenon is reached, the deflection increase within the walls of the structure begins. A characteristic feature of thin-walled composite columns is the ability to continue to carry an axial compressive load after buckling, often several times higher than the load corresponding to the loss of stability [14,15,16,17,18,19,20]. Regarding the above, thin-walled composite columns have a significant reserve of load capacity [21,22,23,24,25,26].

Regarding the failure analysis of thin-walled composite materials, it is important to understand any phenomena that directly contribute to the loss of the load-carrying capacity of the structure [27,28,29,30,31]. Commonly, a few main phenomena that lead to failure are observed. The first of these phenomena is the commonly known damage initiation. From the occurrence of this phenomenon, the permanent failure process of the composite material begins. The above-mentioned phenomenon requires conducting appropriate tests, preferably using both experimental and simulation research methods [25,32,33]. In the framework of fundamental theoretical considerations, the first well-known law which allows the evaluation of the damage initiation of a composite material was the first ply failure theory [34]. The damage initiation, based on the above-mentioned theory, significantly weakens the composite structure. Regarding the experimental evaluation of damage initiation, the commonly applied techniques are based on the possibility of the direct registration of parameters that allow correct interpretation of damage initiation. For this purpose, a frequently used solution is the use of the acoustic emission method. The acoustic emission method (AEM) allows for registering acoustic signals in the form of “elastic waves”, in the context of which it is possible to register such parameters as the number of counts, hits, amplitude or energy [35]. A slightly different situation of damage initiation assessment occurs in the case of numerical simulations. Numerical simulations using the FEM are most often based on the Hashin criterion (damage initiation). The above-mentioned criterion evaluates the damage initiation of the composite material caused by tension/compression of the fibers/matrix [36,37,38]. Generally, failure is interpreted as the loss of the effective cross-sectional area of a structure due to microcracks [39,40]. The damage initiation of the composite structure directly contributed to the permanent damage of the material. As a result of further loading of the composite structure, the damage evolution phenomenon (based on the energy criterion) occurs after damage initiation—according to progressive failure analysis (PFA) [41,42,43,44,45,46]. The other method used to evaluate damage initiation (based on the maximum nominal stress criterion) is analyzing the damage for the occurrence of delamination. The phenomenon of delamination can be considered in the context of both the initiation and evolution of delamination—based on a cohesive zone model (CZM) [47,48,49,50,51]. The above-mentioned method (based on the traction-separation law) allows for rupturing the connection between layers of a composite material [52,53,54].

This paper presents a complex issue, which is the description of the failure mechanism of thin-walled composite columns with open sections [55,56,57,58]. Generally, numerical studies use one of the available failure techniques, while the current work uses a more complex approach to evaluate the failure mechanism. This paper, relative to other thematically similar research papers, primarily provides information and results on the failure phenomenon of thin-walled composite columns using independent numerical models. The novelty of the present work is the use of two independent numerical damage models (PFA and CZM), which allows a better representation of experimental studies. Moreover, the delamination phenomenon was modeled as globally occurring cohesive surfaces between all composite layers. Regarding the above, it is important to apply independent experimental and numerical testing methods in the evaluation of the failure mechanism [59,60,61,62,63].

## 2. Research Methodology

### 2.1. Materials

**Figure 1:**
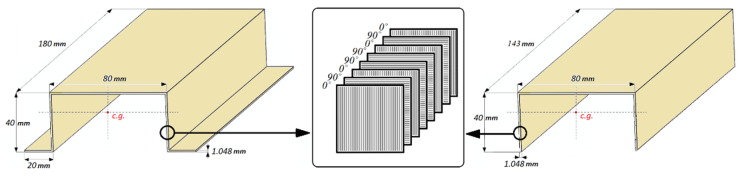
Geometrical parameters of specimens.

The subjects of study were thin-walled composite columns (with top-hat and channel cross-sections). The composite columns were prepared using an autoclave technique (composite structures characterized: low porosity, high repeatability, high strength properties), which was described in the paper [64]. The thin-walled composite columns had similar geometric parameters. Both composite profiles were characterized by the occurrence of the same arrangement of laminate layers [0/90/0/90]_s_. The geometrical parameters of test specimens are presented in Figure 1.

The material properties were determined based on experimental studies using static experimental tests: static tensile test ISO 527, compression test ISO 14126, and shear test ISO 14129, presented in [21]. The material properties, which were determined from the above-mentioned tests, are shown in Table 1 [21].

### 2.2. Experimental Study

**Figure 2:**
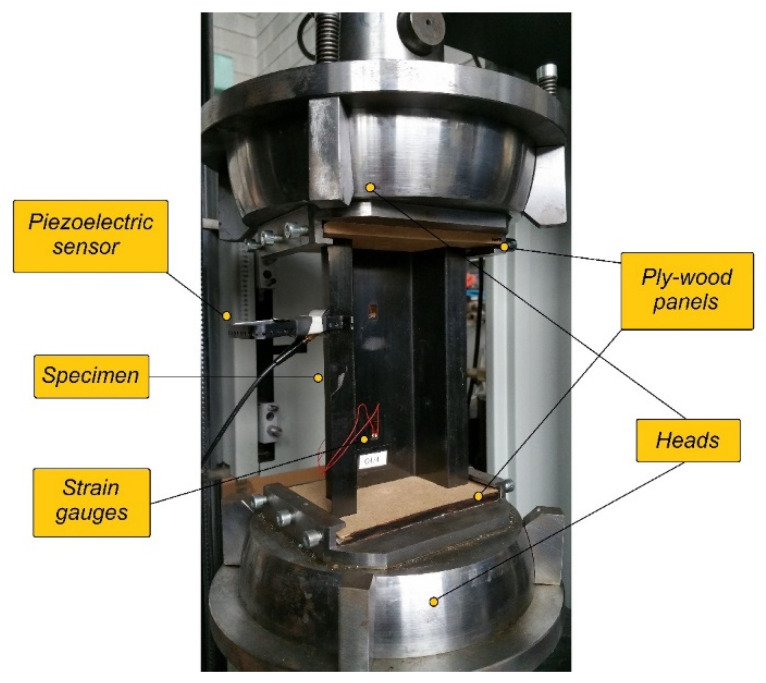
Experimental stand.

Experimental investigations in the context of axial compression were performed up to structural failure. A Zwick Z100 (UTM), as well as an acoustic emission method (AEM) based on the AMSY-5 device, were used. Static compression tests of composite structures were carried out using UTM at room temperature and with a constant crosshead speed of 2 mm/min. The experimental investigations involved the determination of both the post-critical equilibrium paths (representing the relationship between the compressive load and time), as well as selected acoustic emission signals, which allow an in-depth assessment of the phenomena directly accompanying the failure of the composite material. During the experimental tests using UTM, a few parameters were registered: compressive load, shortening of the column, deflection increase (the deflection increase was registered in the perpendicular direction to the web of the structure using strain gauges). In the case of tests based on the acoustic emission method, parameters such as number of counts, hits, energy and amplitude, were recorded. Further analysis of these parameters made it possible to assess the failure of the composite structure [21,24]. The test stand is presented in Figure 2.

The experimental investigations primarily analyzed the loads corresponding to the limit states of the structure, i.e., damage initiation—*P_d_*, delamination—*P_del_* and loss of load-carrying capacity—*P_f_*. A detailed description of the method of determining the limit loads was presented, among others, in the papers [21,54,62].

### 2.3. Numerical Simulations

In the case of numerical simulations carried out in parallel (using the FEM), the buckling modes of the structure were initially determined within the framework of linear calculations of structural stability [65]. The buckling modes were subsequently implemented into the nonlinear structural stability calculations (based on the Newton–Raphson method) [66] for further evaluation of the limit states of the structure (the magnitude of geometric imperfections was 0.05 mm). Numerical simulations were performed using two independent damage models of the composite material (PFA and CZM). Progressive failure analysis was based on damage initiation using the Hashin criterion as well as damage evolution using the energy criterion [67,68]. Hashin’s criterion considers four components of composite material damage initiation: damage due to fiber tension HSNFTCRT (1), fiber compression HSNFCCRT (2), matrix tension HSNMTCRT (3) as well as matrix compression HSNMCCRT (4):

(1) $$F_{f}^{t}={\left(\frac{{\hat{\sigma }}_{11}}{X^{T}}\right)}^{2}+\alpha {\left(\frac{{\hat{\tau }}_{12}}{S^{L}}\right)}^{2}\leq 1,\;{\hat{\sigma }}_{11}\geq 0,$$

(2) $$F_{f}^{c}={\left(\frac{{\hat{\sigma }}_{11}}{X^{c}}\right)}^{2}\leq 1,\;{\hat{\sigma }}_{11}<0,$$

(3) $$F_{m}^{t}={\left(\frac{{\hat{\sigma }}_{22}}{Y^{T}}\right)}^{2}+{\left(\frac{{\hat{\tau }}_{12}}{S^{L}}\right)}^{2}\leq 1,\;{\hat{\sigma }}_{22}\geq 0,$$

(4) $$F_{m}^{c}={\left(\frac{{\hat{\sigma }}_{22}}{2S^{T}}\right)}^{2}+\left[{\left(\frac{Y^{C}}{2S^{T}}\right)}^{2}-1\right]\frac{{\hat{\sigma }}_{22}}{Y^{C}}+{\left(\frac{{\hat{\tau }}_{12}}{S^{L}}\right)}^{2}\leq 1,\;{\hat{\sigma }}_{22}<0,$$

where: *X^T^*, *X^C^*, *Y^T^*, *Y^C^*, *S^L^*, *S^T^* represent longitudinal tensile/compressive strengths, transverse tensile/compressive strengths, longitudinal shear/transverse shear strengths, respectively;$\;{\hat{\sigma }}_{11},\;{\hat{\sigma }}_{22,}\;{\hat{\tau }}_{12,}$ constitute components of stress tensor (effective)—defined in Equation (5), as well as *α*, the contribution of the shear stress (to the fiber tensile).

Generally, the damage initiation phenomenon begins when a minimum of one of the damage initiation components (Equations (1)–(4)) is satisfied. The damage phenomenon can be considered as the loss of effective cross-sectional area of the structure, which is caused by micro-cracks. In order to better describe the damage, a scalar parameter *d* is introduced (the parameter has values in the range from 0 to 1, where 1 denotes the occurrence of damage) [39]. In the framework of the equation describing the effective stress, a scalar damage parameter is considered (5):

(5) $$\hat{\sigma }=\frac{1}{1-d}\sigma ,$$

where: σ represents the Cauchy nominal apparent stress, $\hat{\sigma }$ constitutes the effective stress and *d* is the damage parameter.

The effective stress can be presented using the following equation, representing the relationship between the effective stress and nominal stress, through the damage operator *M* (6):

(6) $$\hat{\sigma }=M\sigma =\left[\begin{array}{ccc} \frac{1}{1-d_{f}} & 0 & 0\\ 0 & \frac{1}{1-d_{m}} & 0\\ 0 & 0 & \frac{1}{1-d_{s}} \end{array}\right]\left\{\begin{array}{c} \sigma_{11}\\ \sigma_{22}\\ \sigma_{12} \end{array}\right\}$$

where *σ_ij_* constitutes the stresses in the *ij* directions; *d_f_*, *d_m_*, *d_s_* represent the parameters of fiber, matrix and shear damage.

The above solution is directly implemented in Abaqus [40]. Using relation (6) and quantitative evaluation of Poisson’s ratio degradation [69], the damaged compliance matrix [*F*] can be expressed by (7):

(7) $$F=\left[\begin{array}{ccc} \frac{1}{\left(1-d_{f}\right)E_{1}} & -\frac{v_{21}}{E_{2}} & 0\\ -\frac{v_{12}}{E_{1}} & \frac{1}{\left(1-d_{m}\right)E_{2}} & 0\\ 0 & 0 & \frac{1}{\left(1-d_{s}\right)G_{12}} \end{array}\right]$$

Then, it is important to specify the corresponding damaged (elasticity) matrix [*C*], which can be represented as (8):

(8) $$C=\frac{1}{A}\left[\begin{array}{ccc} \left(1-d_{f}\right)E_{1} & \left(1-d_{f}\right)\left(1-d_{m}\right)\nu_{21}E_{1} & 0\\ \left(1-d_{f}\right)\left(1-d_{m}\right)\nu_{12}E_{2} & \left(1-d_{m}\right)E_{2} & 0\\ 0 & 0 & A\left(1-d_{s}\right)G_{12} \end{array}\right],$$

It is important to conclude that the parameter *A* included in Equation (8) is described by Equation (9):

(9) $$A=1-\nu_{12}\nu_{21}\left(1-d_{f}\right),$$

Moreover, the damage parameters *d_f_*, *d_m_*, *d_s_*, can be presented as:

(10) $$d_{f}=\{\begin{array}{c} d_{f}^{t},\;if\;{\hat{\sigma }}_{11}\geq 0,\\ d_{f}^{c},\;if\;{\hat{\sigma }}_{11}<0, \end{array}$$

(11) $$\;\;d_{m}=\{\begin{array}{c} d_{m}^{t},\;if\;{\hat{\sigma }}_{22}\geq 0,\\ d_{m}^{c},\;if\;{\hat{\sigma }}_{22}<0, \end{array}$$

(12) $$\;\;d_{s}=1-\left(1-d_{f}^{t}\right)\left(1-d_{f}^{c}\right)\left(1-d_{m}^{t}\right)\left(1-d_{m}^{c}\right)$$

Generally, once the damage initiation phenomenon is satisfied, further loading of the profile contributes to the damage evolution according to the PFA [70]. The phenomenon of damage evolution is based on the energy criterion (energies dissipated during damage for fiber tension *G^c^_ft_* and compression *G^c^_fc_*, as well as matrix tension *G^c^_mt_* and compression *G^c^_mc_* must be defined) and includes five components of composite material damage evolution: damage evolution caused by fiber compression (DAMAGEFC), fiber tension (DAMAGEFT), matrix compression (DAMAGEMC), matrix tension (DAMAGEMT) as well as shear damage (DAMAGESHR).

A second advanced composite failure model was also used in the numerical study. The damage model, known as the cohesive zone model (CZM) based on the traction-separation law, enables complex failure analysis of the composite material. The damage simulation technique based on CZM allows the assessment of the damage phenomenon based on the occurrence of delamination—a permanent rupture of the connection between the composite layers (cohesive surfaces approach). Regarding the description concerning mechanical behavior (in the elastic range of the cohesive layer), the following equation can be used [71]:

(13) $$t=\left\{\begin{array}{c} t_{n}\\ t_{s}\\ t_{t} \end{array}\right\}=\left[\begin{array}{ccc} K_{nn} & K_{ns} & K_{nt}\\ K_{ns} & K_{ss} & K_{st}\\ K_{nt} & K_{st} & K_{tt} \end{array}\right]\left\{\begin{array}{c} \delta_{n}\\ \delta_{s}\\ \delta_{t} \end{array}\right\}=K\delta ,$$

where *t*, *t_n_*, *t_s_*, *t_t_* represent tractions in the cohesive layer; *δ*, *δ_n_*, *δ_s_*, *δ_t_* are the separation displacements of the cohesive layer; *K*, *K_nn_*, *K_ss_*, *K_tt_* constitute the cohesive layer stiffness in global, normal, shear and transverse directions.

As was the case for the PFA-based damage model, the present model considers both the damage initiation and evolution. The damage initiation phenomenon within the CZM model was based on the maximum nominal stress criterion—MAXS. The damage initiation criterion [71] can be represented by Equation (14):

(14) $$max\left\{\frac{\langle t_{n}\rangle }{t_{n}^{0}},\;\frac{t_{s}}{t_{s}^{0}},\frac{t_{t}}{t_{t}^{0}}\right\}=1,$$

where: *t_n_*^0^, *t_s_*^0^, *t_t_*^0^ are the peak values of contact stress, 〈〉 is a Macaulay bracket.

The phenomenon of damage evolution based on the Benzeggagh–Kenane (B–K) criterion [47] was presented using Equation (15):

(15) $$G^{C}=G_{n}^{C}+\left(G_{s}^{C}-G_{n}^{C}\right){\left\{\frac{G_{S}}{G_{T}}\right\}}^{\eta },\;G_{S}=G_{s}+G_{t},\;G_{T}=G_{n}+G_{s}+G_{t},$$

where *G_n_*, *G_s_*, *G_t_* represent fracture energies in normal, first shear and second shear directions; *G^C^* is the mixed-mode fracture energy parameter (critical); *G_n_^C^*, *G_s_^C^*, *G_t_^C^* constitute critical fracture energies in normal, first shear and second shear (directions); *G_S_* is the parameter of total work done by both the shear traction and (relative) displacement components; *G_T_* is the parameter of total work done by normal as well as shear traction; *η* is the parameter of the cohesive property parameter.

Discrete models of composite structures were prepared using the *Continuum Shell* technique. In order to prepare the discrete models, finite elements (FE) of the type *SC8R* were used. Discrete models of the top-hat and channel structures were prepared by modeling each layer of the composite material separately in order to consider the interactions between the layers. This approach made it possible to analyze the failure phenomenon in the context of delamination occurrence. In addition, two non-deformable plate elements (using R3D4 element) were used in the study to support the composite structure. In the case of the discrete model of the top-hat structure with non-deformable plates, the number of finite elements was 13,296 and nodes was 27,342, while in the case of the channel structure with non-deformable plates, the number of finite elements was 8478 and nodes was 17,432.

**Figure 3:**
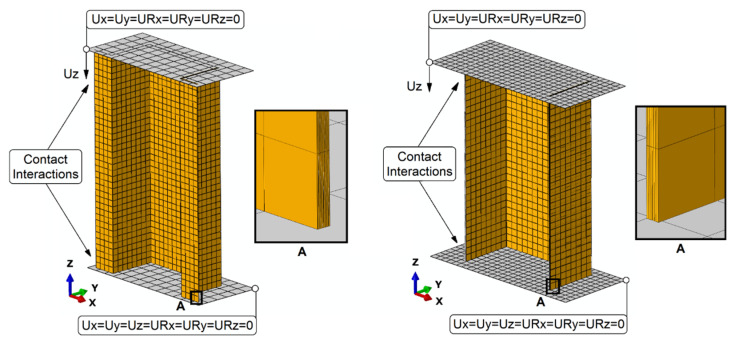
Discrete models with boundary conditions.

Cohesive surfaces were introduced between layers of composite materials using the contact relations. In addition, contact (considering normal and tangential behavior with the friction 0.2) between supports and composite structures was also considered. Regarding the boundary conditions, all relations at reference points coupled to the supports of the composite structures were used (Figure 3).

In the framework of modeling the composite structure, an orthotropic material model was used. The material properties concerning PFA [72], as well as CZM [73], are shown in the papers [21,54].

## 3. Results

### 3.1. Buckling

**Figure 4:**
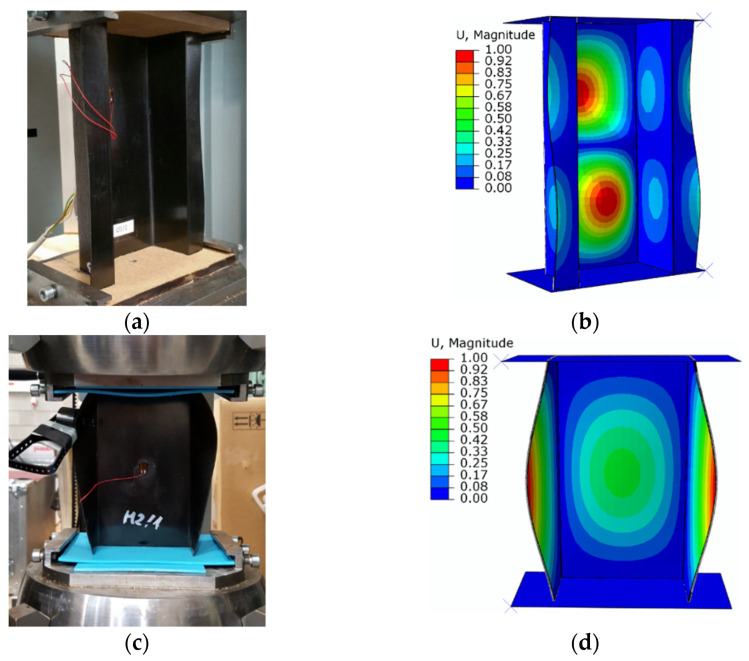
Buckling results: (**a**) EXP of top-hat profile, (**b**) FEM of top-hat profile, (**c**) EXP of channel profile, (**d**) FEM of channel profile.

The experimental investigations and numerical simulations using the FEM initially presented the results of the form of structural stability loss. This paper does not concern the in-depth presentation of the results of critical state tests; only the obtained experimental and numerical forms of buckling are presented, which were used in the further part of the research for the analysis of the post-buckling state. The loss of stability results are shown in Figure 4.

A very high agreement between the forms of loss of structural stability was obtained for experimental studies and numerical simulations using FEM. For both experimental tests and numerical simulations, an identical number of half-waves was obtained in the longitudinal direction of the composite structures. Two half-waves in the longitudinal direction of the top-hat column, and one half-wave for the channel composite column, were observed. The buckling forms obtained by numerical simulations were positively verified by experimental tests. In this paper, the in-depth assessment of the critical state was not of concern since the main focus of the paper was placed on the failure phase of structures made of composite materials [74].

### 3.2. Failure—Experimental Study

**Figure 5:**
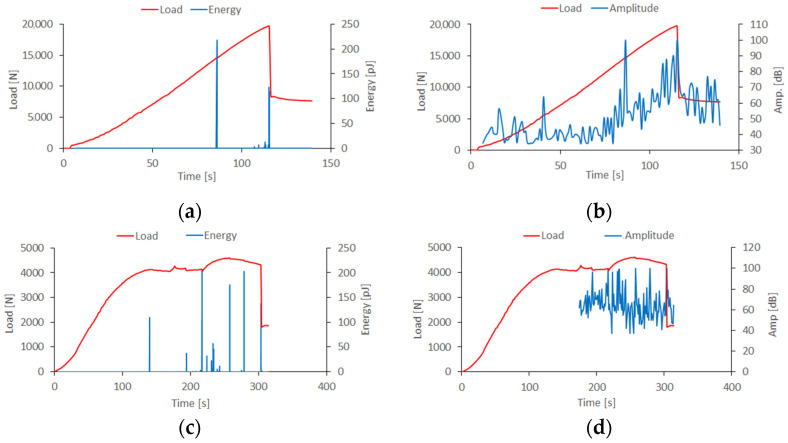
Post-buckling equilibrium paths with selected acoustic emission signals: (**a**) top-hat column energy signal, (**b**) top-hat column amplitude signal, (**c**) channel column energy signal, (**d**) channel column amplitude signal.

The main objective of this study was to present the phenomenon of composite material failure. Regarding the above, post-buckling equilibrium paths describing the character of the behavior of compressed thin-walled composite columns were determined. In the experimental study, post-buckling equilibrium paths were determined with the simultaneous demonstration of selected acoustic emission signals—which allowed for the assessment of the damage phenomenon in a quantitative context. Figure 5 presents the experimental results to evaluate the failure phenomenon of the composite structure.

Experimental studies made it possible to determine limit states based on post-buckling equilibrium paths. Based on the determined characteristics, the approximate values of loads that caused phenomena such as damage initiation, delamination or loss of load capacity of the structure were estimated. Based on the analysis conducted using acoustic emission, the value of the load-causing damage initiation (failure of the first layer of the composite material) was determined. In the case of the tests conducted, the first significant “peak” in the energy signal represented the occurrence of damage initiation, which corresponded to the load designated as *P_d_*. Regarding the above, the damage initiation phenomenon for the top-hat specimen occurred when the energy signal achieved 225 pJ (at 85 s) at a load equal to 14,850 N (Figure 5a). The load-causing delamination occurred beyond 100 s of analysis, where just prior to the loss of load capacity phenomenon, a slight increase in the energy signal (as well as the signal amplitude) began to occur, which was recorded at a load *P_del_* of 18,634 N. The failure load *P_f_*, which represented the highest registered load value on the post-buckling equilibrium path was 19,673 N. In the case of a composite specimen with a channel section, the values of the limit loads were determined in an identical method, then the values of these loads were respectively: *P_d_*, 4134 N; *P_del_*, 4281 N; *P_f_*, 4609 N. The delamination phenomenon for actual specimens was evaluated using macroscopic evaluation and acoustic emission methods. After damage initiation (i.e., the first clear registered energy “spike”), increasing signals, especially amplitudes (Figure 5b,d), indicate the onset of delamination, which occurred just before the loss of load-carrying capacity. The present phenomenon deepened until the end of the experimental tests. Further studies will be conducted using a high-speed camera and a digital microscope.

### 3.3. Failure—Numerical Study

**Figure 6:**
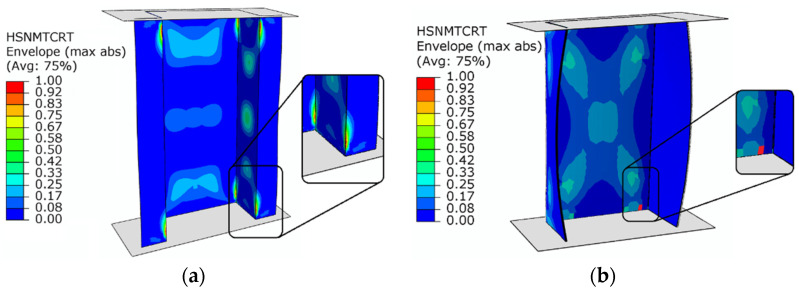
Damage initiation (Hashin criterion—PFA model): (**a**) top-hat column, (**b**) channel column.

In parallel to the experimental studies, numerical simulations using the FEM were carried out in order to evaluate the limit states of structures made of composite materials. The damage initiation was initially evaluated using the Hashin criterion. Using this criterion, it was possible to evaluate whether the damage occurred due to compression or tension of the fibers or matrix of the composite structure. When the damage parameter reached a value of 1, it represented the occurrence of damage initiation. Figure 6 presents the damage initiation based on FEM simulations.

Based on the tests, it was observed that the damage initiation occurred due to the matrix tensile damage of the composite material (HSNMTCRT). The damage initiation was registered at loads: 14,032 N—top-hat specimen and 4222 N—channel specimen.

**Figure 7:**
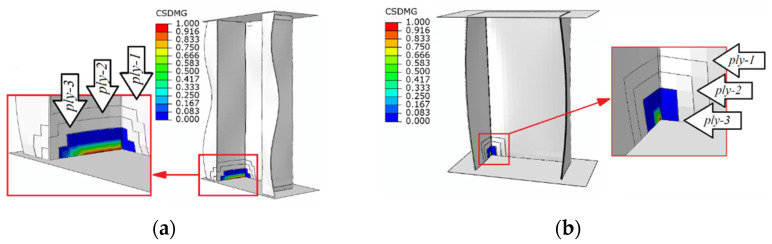
The evolution of delamination (CZM): (**a**) top-hat column, (**b**) channel column.

The next part of the study involved the determination of the loads under which delamination occurs. The initiation of delamination was determined using the criterion maximum nominal stress criterion—CSMAXSCRT. In the case that delamination began, no permanent rupture of the bond between the component layers of the composite material could be observed. Only when the structure was further loaded using the energy criterion did it become possible to show a visible delamination, for which the CSDMG parameter was responsible in the case of the numerical simulations (Figure 7).

The delamination phenomenon occurred mainly on the bottom part of the end section of the composite column (between the third and fourth ply). The delamination occurred at loads: 19,909 N—top-hat specimen, 4774 N—channel specimen.

**Figure 8:**
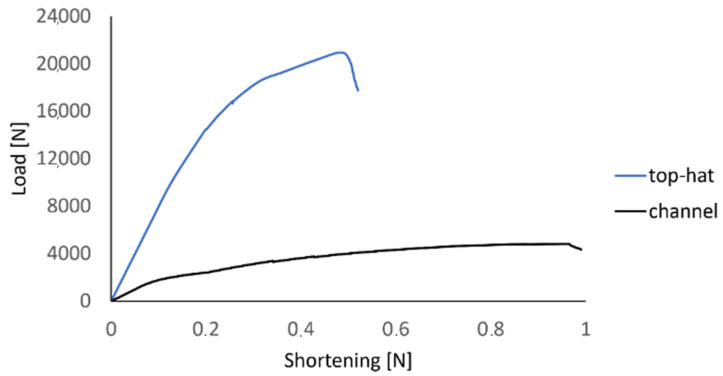
Failure characteristics—results of numerical simulations.

The simultaneous use of two numerical damage simulation techniques, PFA and CZM, allowed for a detailed analysis of the damage phenomenon. In the framework of conducted numerical simulations, the values of loads, which corresponded to the total failure of structures made of composite materials, were estimated (Figure 8).

The loss of load-carrying capacity of the profiles made of composite materials occurred at the following loads: 20,938 N—top-hat column, 4805—channel column.

### 3.4. Failure—Comparison of Experimental and Numerical Results

Based on the research conducted, any results obtained from the quantitative evaluation are summarized in Table 2.

**Figure 9:**
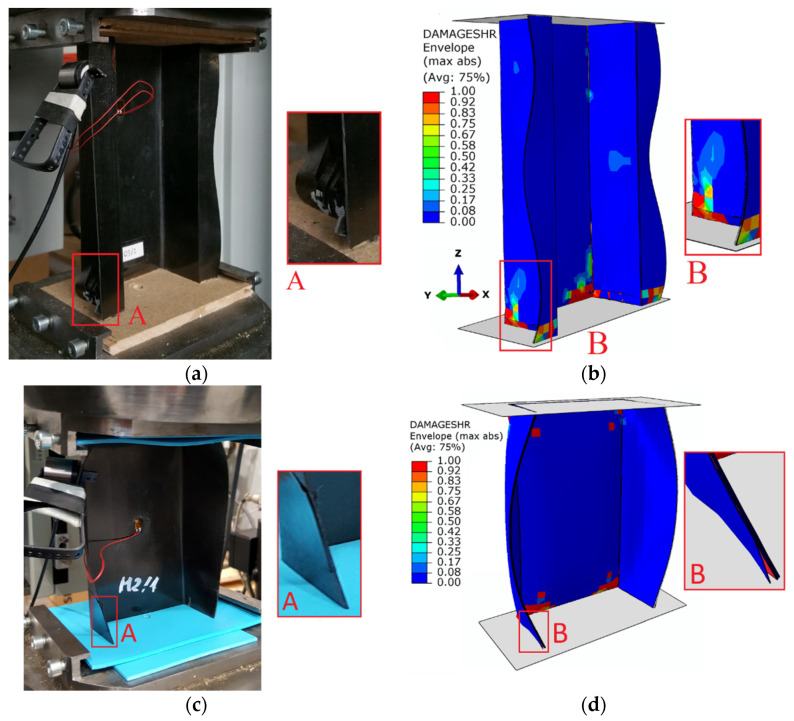
Comparison of failure form: (**a**) EXP of top-hat column, (**b**) FEM of top-hat column, (**c**) EXP of channel column, (**d**) FEM of channel column.

Based on the study, it is demonstrated that the percentage discrepancies between the experimental and numerical results obtained are respectively: 6.04% (failure), 6.4% (delamination), 5.51% (damage initiation)—for top-hat column; as well as 4.08% (failure), 10.33% (delamination), 2.08% (damage initiation)—for channel column. The maximum discrepancy between experimental tests and numerical simulations using the FEM did not significantly exceed 10%. The discrepancies, in terms of limit values, between the top-hat section and channel section structures were significant, which was the reason that top-hat section structures are characterized by several times higher stiffness (construction with top-hat cross-section has two additional flanges). The top-hat structure in relation to the channel structure showed 4.36 times higher failure load, 4.17 times higher load corresponding to the delamination evolution and 3.32 times higher load corresponding to the damage initiation—considering the comparison of results of numerical simulations (experimental results were similar). In order to make a comparison of the failure forms of thin-walled composite columns, the failure forms obtained from experimental tests were compared with the results of numerical calculations—Figure 9.

Based on the comparison of results of the failure forms of the composite material, it was demonstrated that the failure phenomenon occurred in the bottom part of the end sections of the composite columns. Both in the case of the top-hat and channel section structures, a loss of load-carrying capacity phenomenon occurred, which was directly accompanied by delamination. In the case of the channel column, the delamination phenomenon was significantly more apparent; however, in both structures, this phenomenon occurred just before the loss of load-carrying capacity. Both in the context of quantitative and qualitative evaluation, the conducted studies demonstrate high agreement between experimental results and numerical simulations. The research presented in this paper, among others, represents a continuation of the research presented in [75].

The above suggests that the delamination has a direct impact on the weakening of the composite structure in terms of its load-carrying capacity. In the future, more in-depth studies are planned, using other experimental research techniques, including the ARAMIS system (for optical deformation measurement), the VHX-970F digital microscope (for registering progressive damage), and numerical simulation-based XFEM damage modeling technique [76], which allows for failure analysis in the context of composite material fracture. The results were limited to a comparison of experimental studies with numerical simulations [77,78,79,80]. The limitation of the presented experimental studies is only due to the fact that the detailed results of the experimental studies will be presented as a separate scientific paper devoted to a detailed analysis from the point of view of the experimentally captured failure phenomenon.

## 4. Conclusions

The conducted tests, as well as the numerical ones, allowed for evaluating the phenomenon of the failure of columns made of composite structures. The use of interdisciplinary research methods allowed for a thorough consideration of the mechanism of composite material failure. It has been observed that the complex mechanism of failure begins with the damage initiation, which is associated with the failure of the first layer of the composite material, followed by the phenomenon of delamination, immediately preceding the loss of load-carrying capacity of the composite structure. Composite structures with top-hat cross-sections have only two additional external flanges in comparison with structures with channel cross-section, but show more than four times higher failure loads. Based on the conducted research, the following conclusions have been formulated:

- It is possible to assess the failure phenomenon using parallel experimental tests (UTM, AEM) as well as numerical simulations using the FEM;
- The use of several independent failure models within the numerical calculations (PFA, CZM) allow for a detailed evaluation of the composite material failure;
- It is possible to evaluate the phenomena directly contributing to the loss of load-carrying capacity of thin-walled composite columns, such as damage initiation or delamination;
- It is possible to evaluate the load-carrying capacity of compressed thin-walled composite columns having different shapes of cross-sections.

## Figures and Tables

**Table 1:** The composite material properties.

Mechanical		Strength	
*E*_1_ Young’s modulus [MPa]	130,710	*F_T_*_1_ Tensile Strength (0°) [MPa]	1867
*E*_2_ Young’s modulus [MPa]	6360	*F_C_*_1_ Compressive Strength (0°) [MPa]	1531
_V_ Poisson’s ratio	0.32	*F_T_*_2_ Tensile Strength (90°) [MPa]	26
*G*_12_ Kirchhoff modulus [MPa]	4180	*F_C_*_2_ Compressive Strength (90°) [MPa]	214
-	-	*F*_12_ Shear Strength [MPa]	100

**Table 2:** Limit loads.

	Type of Study	*P_d_*—Damage Initiation [N]	*P_del_*—Delamination Damage [N]	*P_f_*—Failure [N]
Top-hat column	EXP	14,850	18,634	19,673
	FEM	14,032	19,909	20,938
Channel column	EXP	4134	4281	4609
	FEM	4222	4774	4805

## Data Availability

Data is contained within the article.
